# Study on FEM Simulation Algorithm of Local Warm Forming of Advanced High-Strength Steel

**DOI:** 10.3390/ma18091900

**Published:** 2025-04-22

**Authors:** Tao Wang, Di Li, Xiao-Kun Wang, Hong-Pai Zhu, Jun-Jie Liu, Ning Jiang, Xiao-Zhi Feng, Shao-Xun Liu

**Affiliations:** 1 School of Transportation and Vehicle Engineering, Shandong University of Technology, Zibo 255000, China; 19811729317@163.com (T.W.); 17753333424@163.com (X.-K.W.); 18179393107@163.com (H.-P.Z.); 13256390277@163.com (J.-J.L.); jiangning@sdut.edu.cn (N.J.); fengxiaozhi@sdut.edu.cn (X.-Z.F.); 2 Rongcheng Compaks New Energy Automobile Co., Ltd., Rongcheng 264300, China; lsx99ht@126.com

**Keywords:** local warm forming, advanced high strength steel, simulation algorithm, fracture, springback

## Abstract

Advanced high-strength steels (AHSSs) are prone to process defects such as fracture and springback during forming operations. Local warm forming technology represents an innovative forming process that applies targeted heating to specific stamping features of high-strength steel blanks. This study focuses on dual-phase steel DP780 as the research material, obtaining mechanical property parameters at various temperatures through uniaxial tensile tests. Based on investigations into temperature-dependent constitutive models and heat-transfer analysis methods, Abaqus VUMAT and UMAT subroutines were developed using Fortran language to establish a local warm forming simulation algorithm that incorporates predictions of fracture failure and springback. A U-shaped component was designed for local warm forming bend-stretch tests, with experimental data compared against results from the developed algorithm. This validation confirmed the algorithm’s capability to accurately predict local warm forming behaviors of U-shaped components. Leveraging the validated algorithm, sensitivity analyses were conducted to examine the influence of local warm forming process parameters on springback, with the response surface methodology employed to quantitatively assess the effects of heating temperature and localized heating zones on springback characteristics.

## 1. Introduction

Advanced high-strength steel, distinguished by high strength and excellent energy absorption capabilities, plays a crucial role in achieving lightweight car bodies. However, steel with higher strength grades exhibits lower elongation and thickness anisotropy indexes [1]. Traditional room temperature (25 °C) forming processes for advanced high-strength steels (AHSSs) have increasingly struggled to meet the complex forming requirements of automotive body components. This challenge has prompted the adoption of elevated-temperature forming techniques to enhance material ductility and formability. While the traditional room temperature (25 °C) forming process and hot forming process enhance formability, they introduce non-negligible challenges that not only increase the process complexity of AHSS-stamped components but also elevate manufacturing costs. Local warm forming technology addresses these limitations by maintaining the material’s original high-strength properties while improving elongation and formability. This innovative approach overcomes the cost barriers and equipment volume constraints associated with traditional thermal forming methods, offering a targeted solution to mitigate springback and fracture issues. As such, it represents an advanced forming technology capable of selectively optimizing material performance during critical forming stages. Advanced high-strength steels (AHSSs) exhibit complex anisotropic and temperature-dependent behaviors during local warm forming. This study adopts the Hill’48 anisotropic yield criterion (Hill, 1948) as the theoretical framework, which accurately captures the planar anisotropy of rolled sheets [2]. Compared to recent higher-order criteria [3], Hill’48 offers superior computational efficiency and industrial applicability. The Swift hardening law (Swift, 1952) is employed to describe strain-hardening behavior, with a modified formulation incorporating both a pre-strain term and temperature-dependent parameters. This model effectively captures nonlinear hardening characteristics in the critical mid-temperature range (200–600 °C), showing 12% higher accuracy than Arrhenius-type models in this regime [4].

The initial research on thermoforming began with lightweight materials for industrial applications, such as magnesium–aluminum alloys and titanium alloys. In 1946, Finch and Wilson experimentally demonstrated that the deep drawing performance of aluminum alloy cups at specific temperatures significantly exceeded that at room temperature (25 °C) [5]. Subsequent investigations by Pepelnjak, T. et al. [6] on DP600 steel sheets revealed an 18% increase in the maximum drawing ratio during cup forming at approximately 300 °C compared to ambient conditions, confirming the enhanced formability of AHSS materials through warm forming processes. Li, D. [7] conducted uniaxial thermal tensile tests and U-shaped stamping simulations on dual phase steel DP780, observing decreasing springback angles with rising forming temperatures.

Building upon these advantages, some researchers proposed integrating warm forming with low-temperature forming by employing localized heating to improve material formability. Bielak, R. et al. [8] utilized laser-assisted localized heating to increase the stamping depth of DP1000 sheets, developing a finite element model in Abaqus for simulation. Karaağaç, İ. et al. [9] performed localized laser-heated V-bending tests on DP800 steel to investigate temperature-dependent variations in springback behavior, mechanical properties, and microstructure. Zhou, J. et al. [10] simulated the localized heated stamping process of boron steel using finite element analysis, while Wang, K. et al. [11] designed a resistance heating system for the localized heating of hot-stamped boron steel, establishing a heat transfer model describing steady-state temperature distributions. Lee, E.H. et al. [12,13] applied infrared localized heating to DP980 steel stampings, demonstrating reduced springback through thermal treatments. Gökhan Küçüktürk, G. et al. [14] conducted localized warm V-bending experiments on Docol 1400 martensitic steel, showing temperature-dependent reductions in springback. Sen, N. et al. [15] systematically studied the influence of localized heat-treatment temperatures on the formability of martensitic 1200 steel during V-bending, optimizing heating parameters through experiments involving temperatures (25 °C, 300 °C, 400 °C and 500 °C), die angles (30°, 60°, 90°, 120°), and holding times (0 and 10 s). Compared to the traditional warm forming technology, the local warm forming technology in the stamping process considers the plate’s exposure to a range of temperatures between the heating zone and the low-temperature zone, spanning from the heating temperature down to the low temperature. However, the pertinent literature predominantly focuses on examining the influence of temperature on the forming properties of high-strength steel sheet material. Recent studies have confirmed the potential of localized heating in suppressing springback, but critical gaps remain: Existing models often decouple thermal and mechanical fields, leading to inaccuracies in predicting multi-physics interactions. There is scant discussion regarding the numerical simulation and analysis method of local warm forming involving thermal coupling. Furthermore, there is a notable absence of integrated thermo-mechanical algorithms to simulate the local warm forming process, which causes obstacles to the research and industrial application of the local warm forming process.

The core value of this work lies in bridging the gap in multi-physics simulation algorithms for local warm forming. By proposing a thermo-mechanical coupling framework and synergistic defect prediction model, it provides a high-precision, efficient digital tool for forming AHSS complex components. Compared to existing studies, this algorithm achieves breakthroughs in prediction accuracy, industrial applicability and multi-field coupling completeness.

This study conducted uniaxial tensile tests on DP780 steel at varying temperatures (25–600 °C) to characterize its mechanical parameters. Fortran-based user-defined material subroutines (VUMAT and UMAT) were developed for Abaqus to establish a simulation algorithm for the local warm forming of sheet metals. The algorithm’s reliability was validated through comparative analysis of simulation results against experimental data. Subsequently, based on the validated algorithm, investigations were performed to analyze the sensitivity of springback to local warm forming process parameters. Response surface methodology was employed to quantitatively evaluate the influence of heating temperature and localized heating zones on springback characteristics.

## 2. Material Properties and Constitutive Model

In this study, dual-phase steel DP780 produced by Shanghai Baosteel company (Shanghai, China) was selected. The chemical composition of dual-phase steel DP780 plate material is shown in Table 1.

### 2.1. Yield Criterion

After rolling, metallic materials exhibit obvious anisotropic characteristics [16]. The Hill’48 anisotropic yield criterion accurately characterizes the anisotropic properties of sheet metals [17,18]. In this paper, the Hill’48 anisotropic yield criterion was utilized to establish a simulation model for dual-phase steel DP780. The expression is shown in Equation (1):

(1) $$\bar{\sigma }=\sqrt{F{\left(\sigma_{y}-\sigma_{z}\right)}^{2}+G{\left(\sigma_{z}-\sigma_{x}\right)}^{2}+H{\left(\sigma_{x}-\sigma_{y}\right)}^{2}+2L\sigma_{\mathrm{yz}}^{2}+2M\sigma_{\mathrm{zx}}^{2}+2N\sigma_{\mathrm{xy}}^{2}}$$

where $\bar{\sigma }$ is the equivalent stress, $\sigma_{i}$ represents the component of the Cauchy stress tensor, and the parameters *F*, *G*, *H*, *L*, *M*, and *N* are anisotropic constants that can be converted from the anisotropy index *r* (also referred to as the plastic strain ratio). The *r* values along three orientations—0°, 45°, and 90° relative to the rolling direction—were determined through uniaxial tensile tests, denoted as *r*_0_, *r*_45_, and *r*_90_, respectively. The *r*-value is shown in Table 2 [19]. The literature [20,21,22] verified the stability of anisotropy parameters in DP steel at varying temperatures through experiments and simulations. The anisotropy parameters of the studied dual-phase steel were provided by the steel company, as shown in Table 3. Since the impact on computational accuracy is negligible, this paper adopts the data listed in Table 3 for all simulation analyses of dual-phase steel DP780 across different temperatures.

### 2.2. Unidirectional Tensile Test

**Figure 1:**
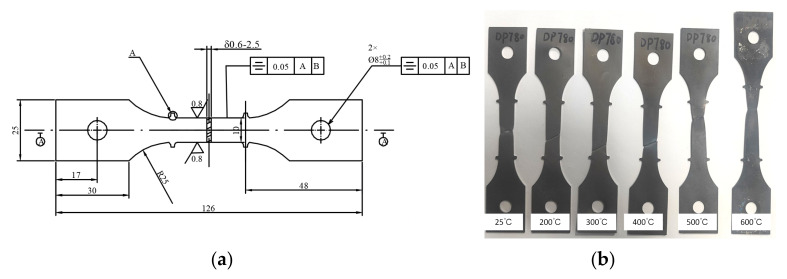
(**a**) Dimension of the tensile specimen and (**b**) tensile specimens at different temperatures.

In order to determine the mechanical properties of the sheet at different temperatures, a WDW–20D material performance testing machine was employed to conduct uniaxial tensile tests on dual-phase steel DP780 at 25 °C, 200 °C, 300 °C, 400 °C, 500 °C and 600 °C. The dimensions of uniaxial tensile specimens are shown in Figure 1a, and the tensile specimens at different temperatures are shown in Figure 1b.

**Figure 2:**
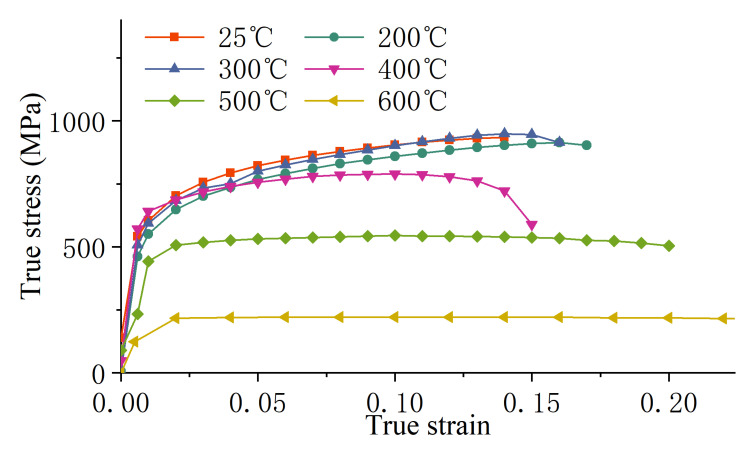
True stress–strain curves of DP780 dual-phase steel sheet at different temperatures.

The true stress–strain curve of dual-phase steel DP780 sheet material at different temperatures is shown in Figure 2.

### 2.3. Stress–Strain Relationship

Swift [23] investigated the influence of the initial yield strain on the material’s constitutive behavior. To develop a more appropriate material model, this study adopts a modified Swift hardening law [24] to characterize the mechanical properties of dual-phase steel sheet material. The mathematical formulation of this enhanced hardening model is provided in Equation (2):

(2) $$\sigma =K(\varepsilon_{0}+{\bar{\varepsilon }}_{p}{)}^{n}\times (1-c\times {\bar{\varepsilon }}_{p})$$

where *K* is the hardening coefficient, $\varepsilon_{0}$ is the pre-strain, *n* is the hardening index, $\varepsilon_{p}$ is the equivalent plastic strain, and *c* is the softening parameter.

Using the real stress–strain curves and Equation (2), the hardening factor *K* and hardening index *n* of the plate at different temperatures were determined. After fitting analysis using Origin software (Origin 2018), the variation rules for the *K* value, *n* value and *c* value are shown in Equations (3), (4), and (5), respectively:

(3) $$K=\left\{\begin{array}{ll} \;1374.6-1.4T+4.5E^{-3}T^{2} & T\leq 300\;{}^{\circ}C\\ -7940+55.6T-8.2E^{-2}T^{2} & 300\;{}^{\circ}C\leq T\leq 450\;{}^{\circ}C\\ 5211.4-13.73T+9E^{-3}T^{2} & 450\;{}^{\circ}C\leq T\leq 600\;{}^{\circ}C \end{array}\right.$$

(4) $$n=\left\{\begin{array}{cc} \;\;\;\;\;\;0.17 & T\leq 250\;{}^{\circ}C\\ \;2.91-2.853E^{-2}T+9.78E^{-5}T^{2}-1.1E^{-7}T^{3} & 250\;{}^{\circ}C\leq T\leq 450\;{}^{\circ}C\\ 0.74-2.31E^{-3}T+1.823E^{-6}T^{2} & 450\;{}^{\circ}C\leq T\leq 600\;{}^{\circ}C \end{array}\right.$$

(5) $$c=\left\{\begin{array}{cc} \;0 & \;\;25\;{}^{\circ}C\leq T\leq 500\;{}^{\circ}C\\ \;\;1 & \;\;\;\;450\;{}^{\circ}C\leq T\leq 600\;{}^{\circ}C \end{array}\right.$$

where *K* is the hardening coefficient, *n* is the hardening index, and *c* is the softening parameters.

**Figure 3:**
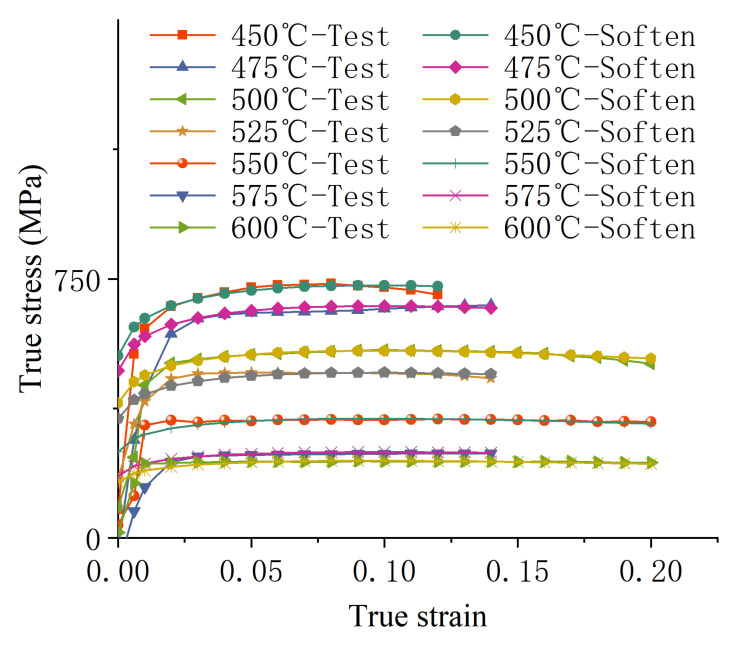
Comparison between the modified fitting results and experimental results for “Softening Phenomenon”.

By substituting the parameters *K*, *n*, and *c* at different temperatures into Equation (2) and comparing them with test results—as shown in Figure 3, where the test curves represent experimental data and the Soften curves depict stress–strain relationships incorporating the “softening phenomenon”—it can be observed that the proposed modified Swift model effectively describes the softening phenomenon exhibited by the sheet material at higher temperatures within the warm forming range.

### 2.4. Temperature-Related Material Parameters

The physical parameters required for partial simulations of the dual-phase steel DP780 sheet were determined using JMatPro10.0, a specialized software for phase diagram calculations and material property simulations in metallic systems. This multifunctional tool enables the computation of multi-component equilibrium phase diagrams and various thermomechanical properties by inputting elemental compositions and employing its integrated material science algorithms [25,26].

By inputting the elemental composition and mass fractions of dual-phase steel DP780 into JMatPro’s steel material analysis module, the software’s built-in physical and thermophysical property calculation algorithms enable the systematic determination of temperature-dependent material attributes.

For local temperature forming simulation analysis in Abaqus software (Abaqus 14.0), it is necessary to define two types of physical parameters of the DP780 two-phase steel sheet. The first is the physical parameters of elastoplastic mechanics, including density, Young’s modulus and Poisson’s ratio; the second is the thermal physical parameters related to heat transfer, including the thermal expansion coefficient, thermal conductivity and specific heat. Both parameters change with temperature. By obtaining the data of the above material parameters with the temperature through JMatPro and defining the relevant material properties in Abaqus software and the material constitutive model subroutine, the stress–strain relationship of the DP780 two-phase steel sheet at different temperatures can be accurately described.

**Figure 4:**
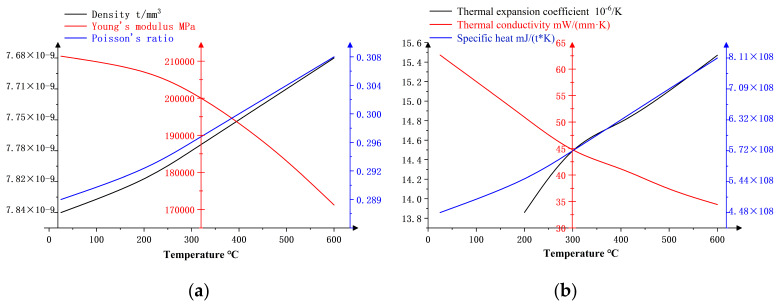
(**a**) Elasto-plastic mechanical parameters; (**b**) thermophysical parameters.

The variation rules of each material parameter for the dual-phase steel DP DP780 material with the temperature are shown in Figure 4.

## 3. FEM Algorithm

### 3.1. Fracture Criteria

Wierzbicki [27] proposed the Modified Mohr–Coulomb (MMC) fracture toughness criterion, which is extensively utilized in predicting shear-type fractures in advanced high-strength steels. Its expression is shown in Equation (6):

(6) $$D=\int_{0}^{{\bar{\varepsilon }}_{f}}\frac{d{\bar{\varepsilon }}_{p}}{{\hat{\varepsilon }}_{f}\left(\eta ,\bar{\theta }\right)}=C=1$$

where *D* is the damage index, ${\hat{\varepsilon }}_{f}\left(\eta ,\bar{\theta }\right)$ is the weight coefficient, $d{\bar{\varepsilon }}_{p}$ is the equivalent plastic strain increment, $\left(\eta ,\bar{\theta }\right)$ is the current incremental step stress state, *C* is the damage index at the moment of fracture, and $\overset{—}{\varepsilon f}$ is the equivalent plastic strain at the moment of fracture.

Bai and Wierzbicki [28,29] established the fracture envelope function, which is shown in Equation (7):

(7) $${\hat{\varepsilon }}_{f}={\left\{\frac{K}{c_{2}}\left[c_{3}+\frac{\sqrt{3}}{2-\sqrt{3}}\left(1-c_{3}\right)\left(\sec\frac{\bar{\theta }\pi }{6}-1\right)\right]\left[\sqrt{\frac{1+c_{1}^{2}}{3}}\cos\frac{\bar{\theta }\pi }{6}+c_{1}\left(\eta +\frac{1}{3}\sin\frac{\bar{\theta }\pi }{6}\right)\right]\right\}}^{-\frac{1}{n}}$$

where η is the stress triaxiality, $\overset{—}{\theta }$ is the Rod angle, *K* is the hardening coefficient, *n* is the hardening index, and *c* is the parameter to be determined.

Our research team previously published work [30] that investigated the correlation between the MMC fracture failure criteria parameters *c*_1_, *c*_2_, *c*_3_ and the sheet material temperature. Temperature-dependent relationships for these parameters were established across the range of 25 °C to 600 °C, as expressed in Equations (8)–(10):

(8) $$c_{1}=a_{1}+b_{1}e^{\frac{T}{m_{1}}}$$

(9) $$c_{2}=a_{2}+b_{2}e^{\frac{T}{m2}}$$

(10) $$c_{3}=a_{3}T+b_{3}$$

where *T* is the temperature, *a*_i_, *b*_i_, *m*_i_ are the values of the fitted coefficient, and *c*_1_, *c*_2_, and *c*_3_ are the parameters to be determined by the MMC criterion. Substituting the aforementioned law into Equation (8) yields the MMC fracture criterion for fracture prediction, taking into account the temperature effect as utilized in this paper.

### 3.2. Heat Transfer Analysis

**Figure 5:**
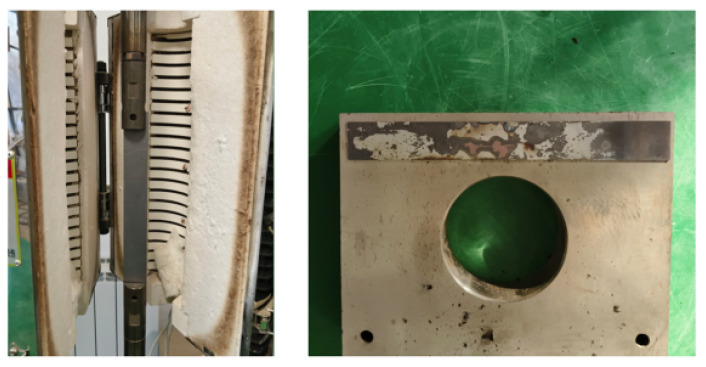
Heat exchange test device for sheet and mold material.

During local warm forming of dual-phase steel sheet material, both mechanical loading and thermal transfer processes interact significantly. Heat transfer alters the sheet temperature distribution, which subsequently affects its mechanical properties. Based on principles of heat transfer theory, this study employs the heat transfer analysis module in Abaqus software to develop a thermal simulation model for the local warm forming process. To validate the accuracy of the Abaqus-based thermal model in predicting local forming temperatures, real-world heat transfer data from physical experiments are required for comparison. A dedicated heat transfer experiment was designed to characterize thermal interactions between the sheet and forming die, with a corresponding simulation model established in Abaqus. The numerical and experimental temperature–time response curves were compared to verify the computational fidelity of the Abaqus heat transfer module. The experimental setup for thermal characterization is illustrated in Figure 5.

After heating the sheet material to a predetermined temperature, it was positioned on a die, and the temperature variation at the center of the sheet over time following contact with the die was recorded to obtain heat transfer data between the sheet material and the die.

Prior to establishing the simulation model, the heat transfer process of the sheet material was analyzed based on heat transfer theory. This analysis encompasses three distinct mechanisms: Internal heat conduction within the sheet material from the heated zone to the unheated zone; interfacial heat conduction between the sheet material and the die; and convective heat transfer and thermal radiation between the heated sheet material and the surrounding ambient air.

Following the analysis of the heat transfer process, a geometric model of the sheet material and dies was established based on actual dimensions, with corresponding material properties assigned. The material properties of the sheet were defined according to the temperature-dependent parameters described in Section 2. Due to the significantly larger volume of the dies compared to the sheet material in the heat transfer experiment, their temperature exhibited a negligible elevation. It was therefore inferred that the material properties of the dies remained temperature-independent, and thus constant values were assigned to all die material attributes.

**Figure 6:**
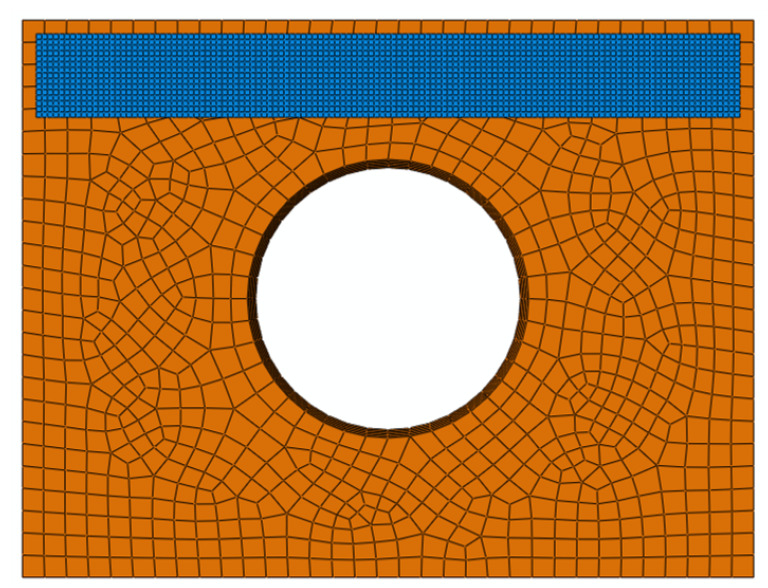
Heat transfer model meshing.

The thermal mesh for the model was specifically configured for the heat transfer analysis step. The sheet material was discretized using DS4 thermal shell elements with a grid size of 2 mm × 2 mm, while the dies were meshed with DC3D8 thermal hexahedral elements at a coarser resolution of 10 mm × 10 mm, as illustrated in Figure 6. In the loading module, a predefined temperature field matching the measured sheet temperature upon removal from the furnace was applied to the sheet surface. During experimental validation, the ambient temperature was recorded as 25 °C. Consequently, a fixed temperature field of 25 °C was imposed on both the die surfaces and the thermal boundary conditions at the die periphery and base.

**Figure 7:**
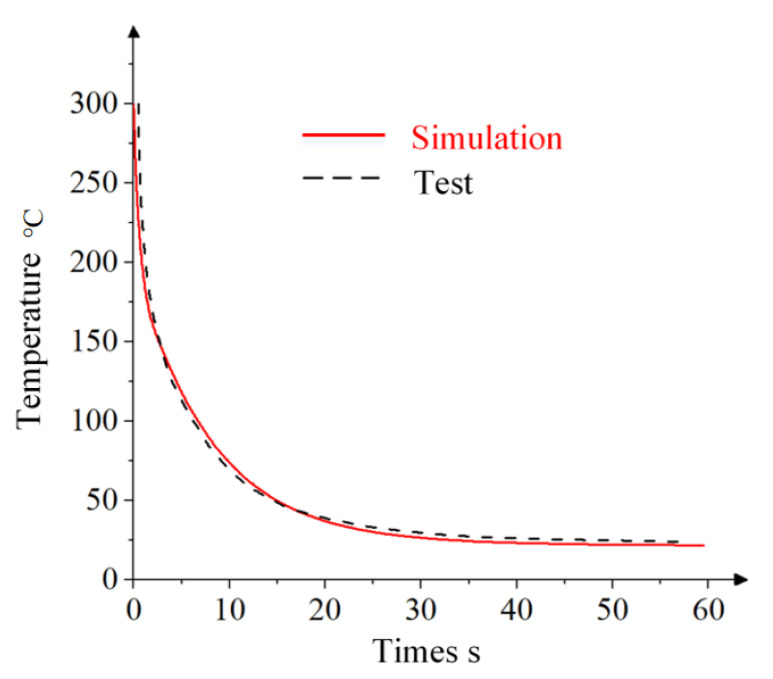
Precision comparison of heat transfer model.

Following the completion of the simulation, a cell at the central position on the sheet was selected, and the temperature-versus-time curve of the cell was output, as shown in Figure 7.

The temperature distribution predicted by the heat transfer analysis model exhibited a maximum deviation of less than 2 °C compared to experimental measurements, demonstrating a high accuracy. This validates that the Abaqus-based heat transfer model effectively simulates the actual temperature evolution during the local warm forming process.

### 3.3. Algorithm for Local Warm Forming Simulation

The local warm forming simulation algorithm has been constructed utilizing the Abaqus VUMAT explicit algorithm subroutine, programmed in the Fortran language. The flow of this algorithm is outlined as follows:

(1) The material parameters and initial temperature field localized in the sheet are set in Abaqus software.
(2) Heat transfer analysis is carried out using the established heat transfer analysis model to obtain the temperature distribution of the sheet.
(3) The VUMAT subroutine reads the updated temperature, material parameters, and strain increment matrix, and calculates the plastic strain increment in this analysis step by computing the test stress based on the strain increment according to the generalized Hooke’s law.
(4) Determining whether the material is in the yielding stage, and if yielding does not occur, the stress is updated based on the value of the test stress.
(5) If yielding occurs, calculating the equivalent plastic strain is performed and the stresses in each direction are updated, and the equivalent plastic strain and each plastic strain component are recorded in the user state variables.
(6) Mechanical parameters, such as the equivalent fracture strain, Lode angle parameter, and stress triaxiality, are obtained by calculating the updated stress state.
(7) Determining whether the corresponding unit is ruptured using the MMC fracture criterion that took into account the effect of temperature.
(8) Updating energy, purely elastic analyses step update internal energy, elastic–plastic analyses step update internal energy with inelastic dissipation energy.
(9) End.

**Figure 8:**
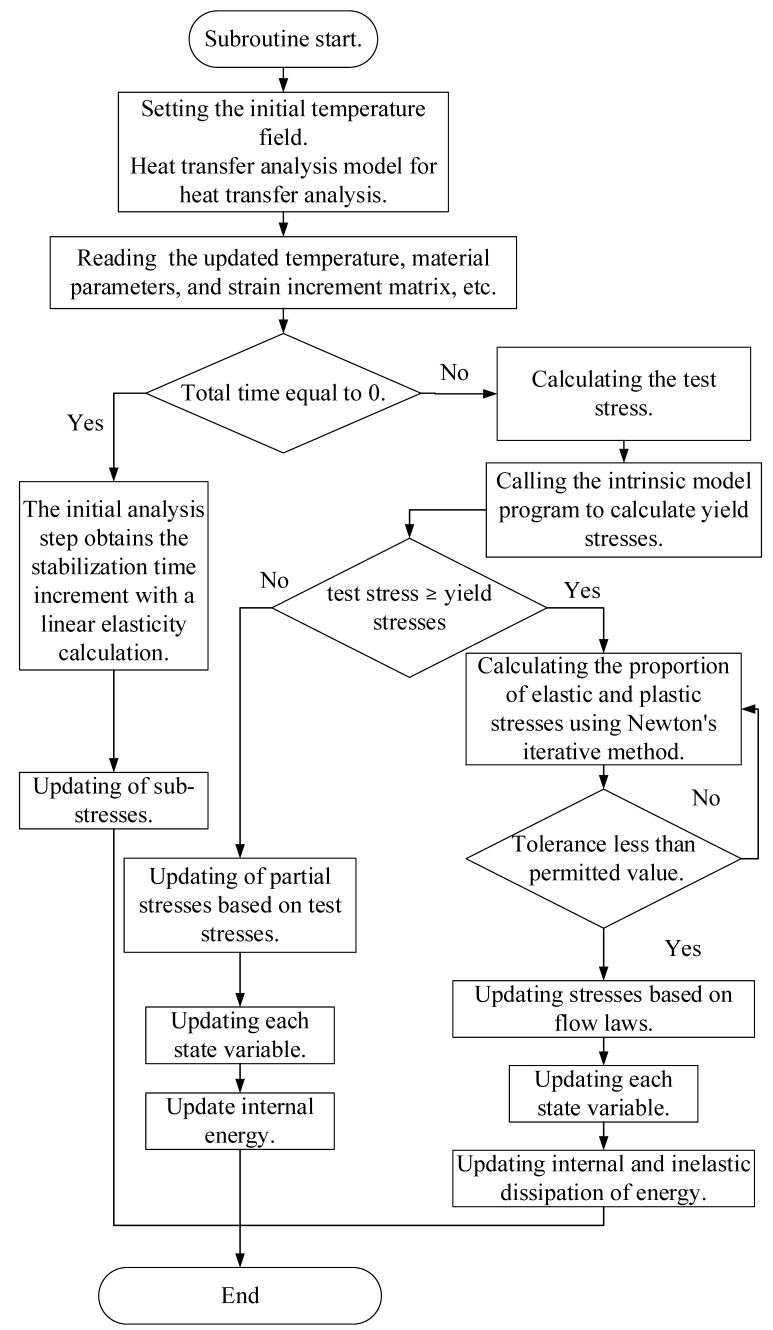
Subroutine flow chart.

Figure 8 shows the subroutine flow chart of the constitutive model subroutine.

### 3.4. Springback Algorithm

The simulation of sheet springback demands the application of a static implicit algorithm, with the springback algorithm being executed through the development of an Abaqus UMAT subroutine in Fortran programming language. The flow of this algorithm is outlined as follows:

(1) At the end of the forming simulation, a restart is set up to start the springback analysis.
(2) The initial temperature field localized in the sheet is set in Abaqus software. Heat transfer analysis is carried out using the established heat transfer analysis model to obtain the temperature distribution of the sheet.
(3) After reading the temperature on each grid after updating, the UMAT subroutine calculates the elastic parameters and elastic Jacobi matrix for each cell based on the relationship between the elastic modulus and Poisson’s ratio and temperature, calculates the stresses based on the strains, and reads the value of each strain stored in the state variables from the previous step.
(4) The UMAT subroutine incorporates the current temperature into Equations (3)–(5), calculates the yield stress of each unit at the current temperature by combining the current equivalent plastic strain, and substitutes it into the Swift hardening criterion. The deviatoric stress is calculated, and the equivalent stress is calculated according to Equation (1).
(5) Determining the yielding situation, if yielding does not occur, then go to the purely elastic incremental step of the calculation process to calculate the relevant variables, and the equivalent plastic strain is 0.
(6) If yielding occurs, the process of calculating each stress–strain value, equivalent plastic strain and updating the elastic–plastic Jacobi matrix is carried out in the elastic–plastic incremental step.
(7) Each state variable is updated and stored.
(8) End.

**Figure 9:**
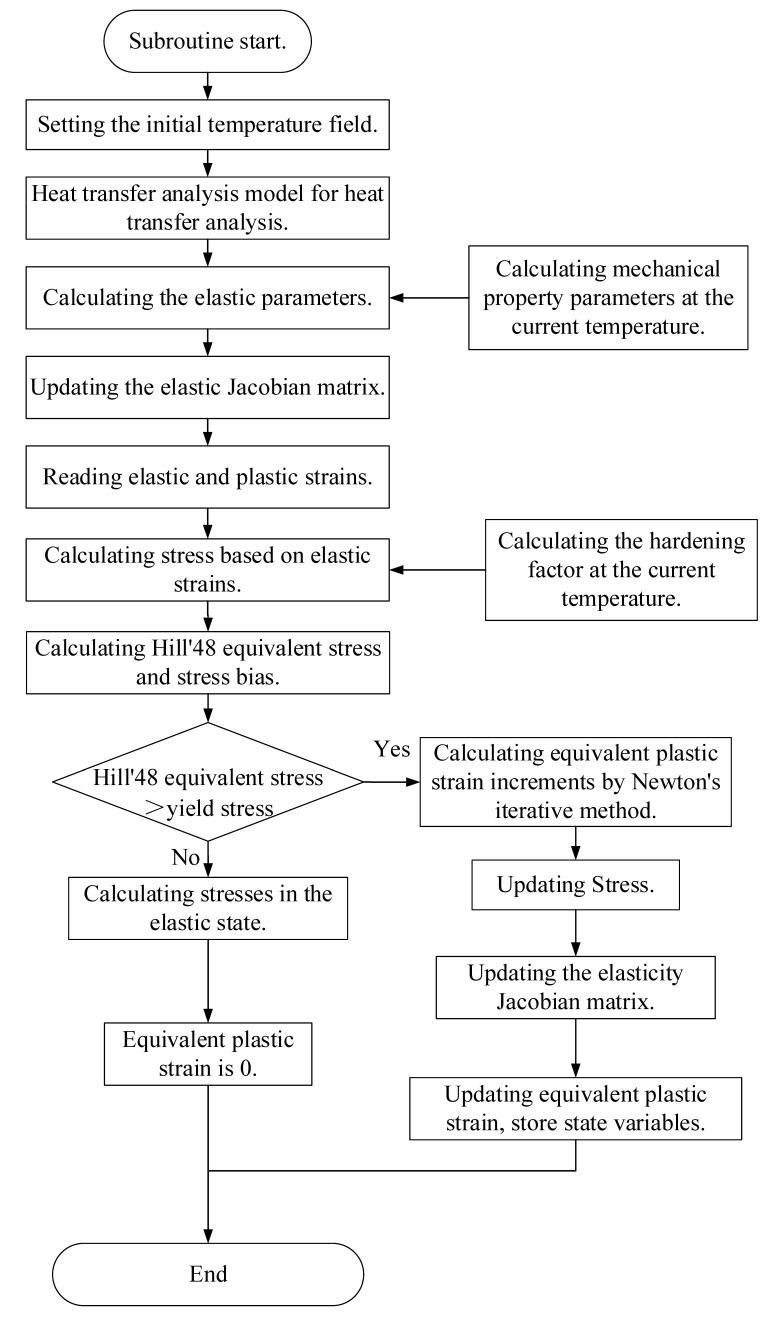
Flowchart of UMAT subroutine for local warm forming springback simulation.

Figure 9 shows the subroutine flow chart of the constitutive model subroutine:

## 4. Verification

### 4.1. Forming Test

**Figure 10:**
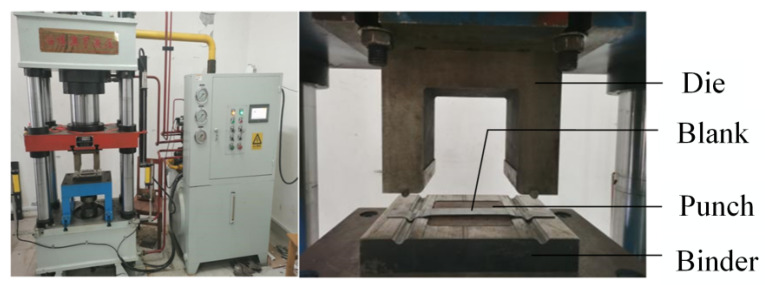
CNC four-column hydraulic press.

**Figure 11:**
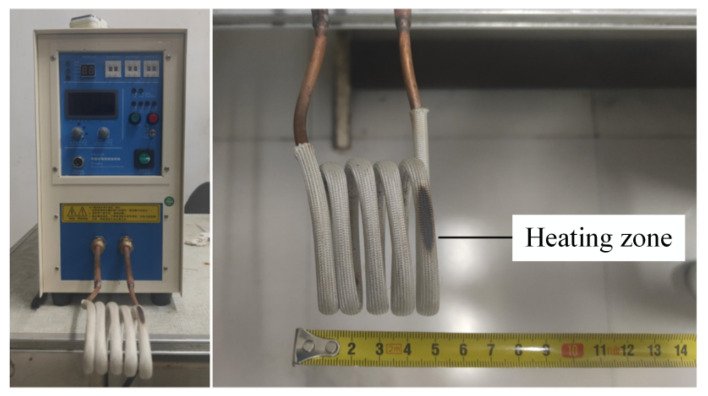
Heating equipment and heating coils.

**Figure 12:**
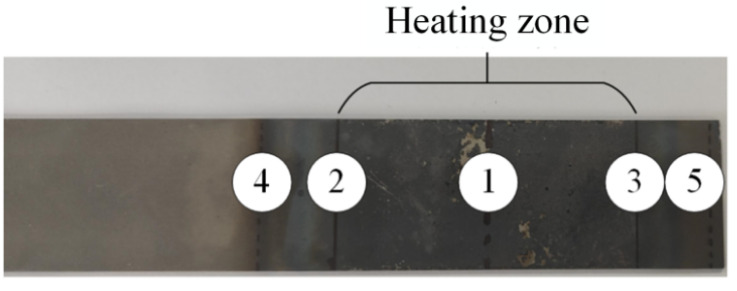
Localized heating zone for thermal forming.

A 160-ton CNC four-column hydraulic press, model YZ32-160S (Zibo Aoheng Hydraulic Machinery Co., Zibo, China), was employed to conduct local warm forming bending tests on U-shaped parts. The test equipment is shown in Figure 10 and Figure 11. The local warm forming heating zone is shown in Figure 12, and the heating zone is located between points 2 and 3 with a width of 60 mm. The detailed test process parameters are shown in Table 4.

**Figure 13:**
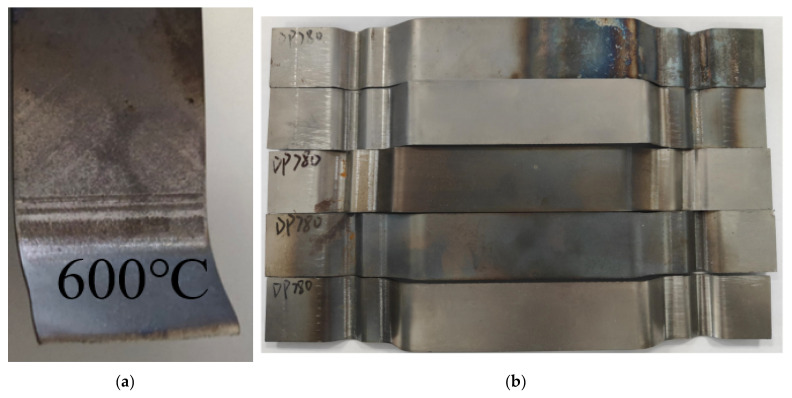
(**a**) Local warm forming fracture test result and (**b**) local warm forming springback test results.

The results of the local warm forming tension bending test of U-shaped parts are shown in Figure 13. The specimen that ruptured during this test was selected as the result of the local warm forming fracture test. It is shown in Figure 13a, with a depth of stamping of 9.4 mm, and the result of the local warm forming springback test is shown in Figure 13b.

**Figure 14:**
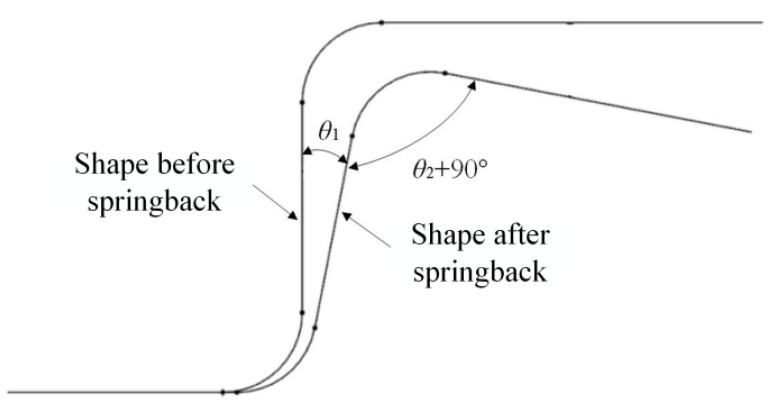
Evaluation criteria for springback of U-shaped parts.

Considering the characteristics of springback in U-shaped parts, particularly when focusing on the sidewall angle and flange angle, this paper follows the evaluation indexes for both the sidewall angle and flange angle (*θ*_1_ and *θ*_2_, as shown in Figure 14). These indices are derived from the results of tensile bending tests conducted on U-shaped parts, alongside the outcomes of simulations, and are utilized to assess the sidewall angle and flange angle.

### 4.2. Simulation

**Figure 15:**
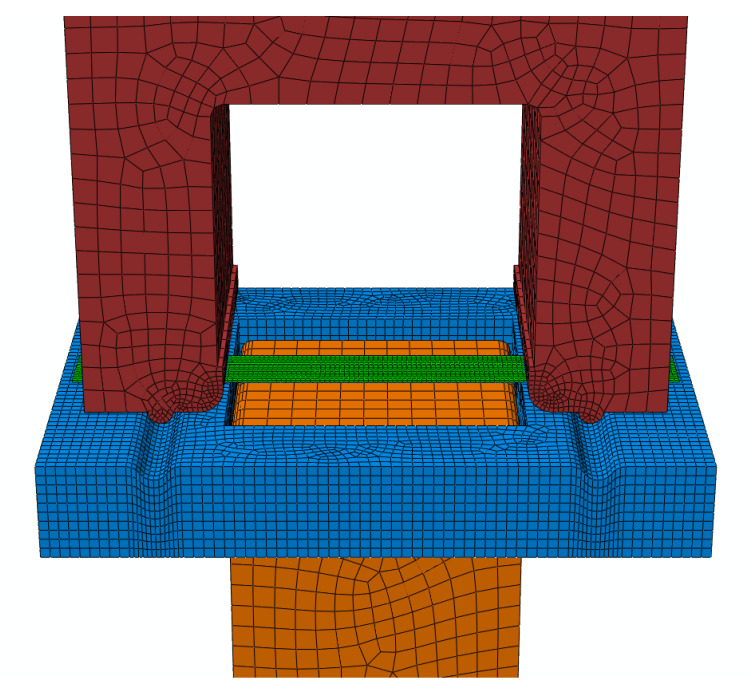
U-shaped parts bending model.

A finite element model for the local warm forming simulation of a U-shaped stretch-bend specimen was established in Abaqus based on the actual dimensions of the die and blank, as shown in Figure 15. The blank was modeled as S4RT thermally coupled shell elements with 2016 nodes and 1875 elements, featuring a uniform mesh size of 2 mm × 2 mm and a constant thickness of 1 mm. The tooling system consisted of C3D8RT thermally coupled hexahedral elements with 5 mm × 5 mm base dimensions. The binder contained 5685 nodes and 5724 elements, the die comprised 1179 nodes and 1040 elements, while the punch was discretized with 1704 nodes and 1610 elements. Local mesh refinement was implemented in critical regions, including the drawbead areas and punch fillet zones, to ensure computational accuracy.

**Figure 16:**
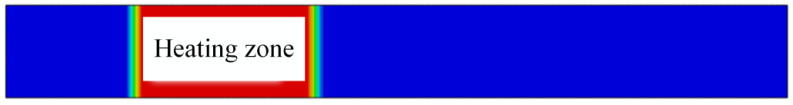
The heating zone of U-shaped part.

Firstly, the VUMAT user material subroutine was embedded into the Abaqus software to facilitate the stamping process. The heating zone is shown in Figure 14 with a width of 60 mm; the temperature of the heating zone is 600 °C, and the temperature of the unheating zone is 25 °C. The simulation results are shown in Figure 16.

**Figure 17:**
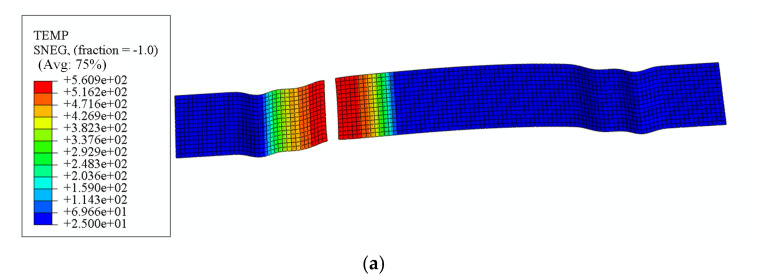

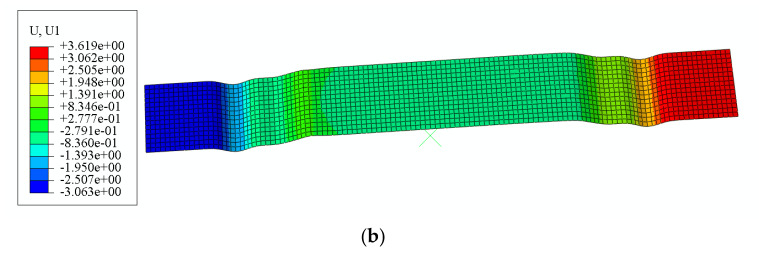
(**a**) Temperature distribution at the end of forming; (**b**) the horizontal displacement of the sheet at the moment before fracture.

Figure 17a illustrates the temperature distribution at the conclusion of the forming process, while Figure 17b depicts the horizontal displacement of the sheet material immediately prior to fracture. The absolute value of this displacement serves as a metric for evaluating the material feed. Fracture occurred in the heated zone of the sheet material, with a simulated stamping depth of 9.2 mm. This behavior arises from the lower stress–strain curve in the heated region, which promotes enhanced plastic deformation. During concurrent deformation on both sides of the U-shaped component, the reduced strength and diminished stress generation in the heated zone caused it to undergo tensile stretching dominated by the adjacent unheated region. The discrepancy between simulated and experimental fracture depths at rupture initiation was 2.1%, confirming that the proposed local warm forming simulation algorithm achieves an accurate prediction of fracture under localized thermal forming conditions.

**Figure 18:**
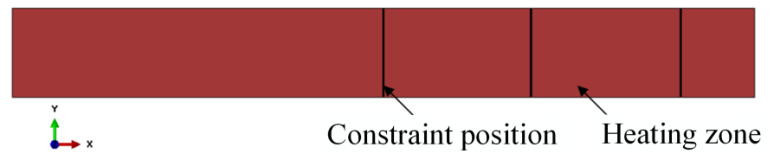
The constraint position and heating zone.

Following the completion of the stamping process, the constitutive model UMAT user material subroutine was embedded into Abaqus software. Specific modules of the software were reconfigured, with Figure 18 illustrating the heating zone implemented in the local warm forming springback simulation. Upon finalizing these settings, springback analysis was conducted.

**Figure 19:**
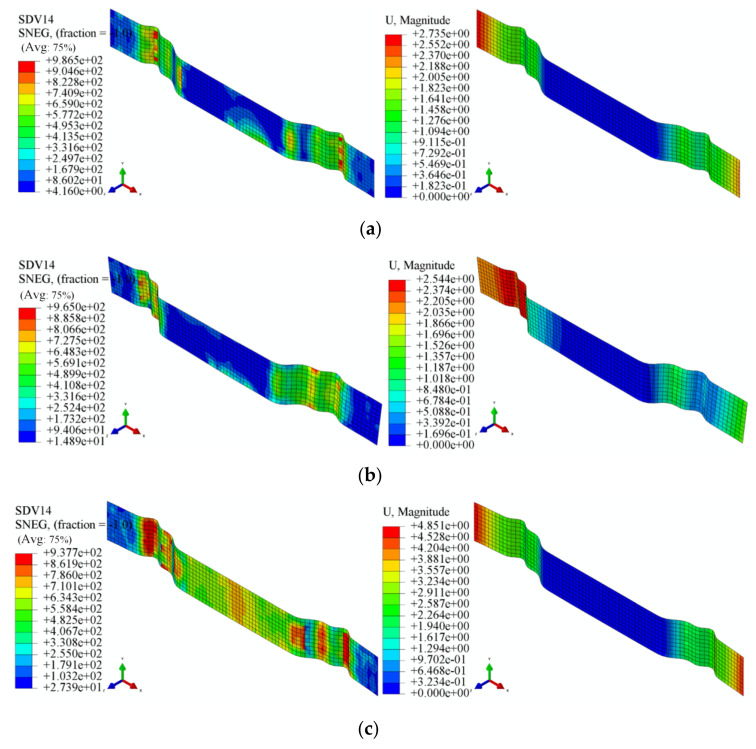

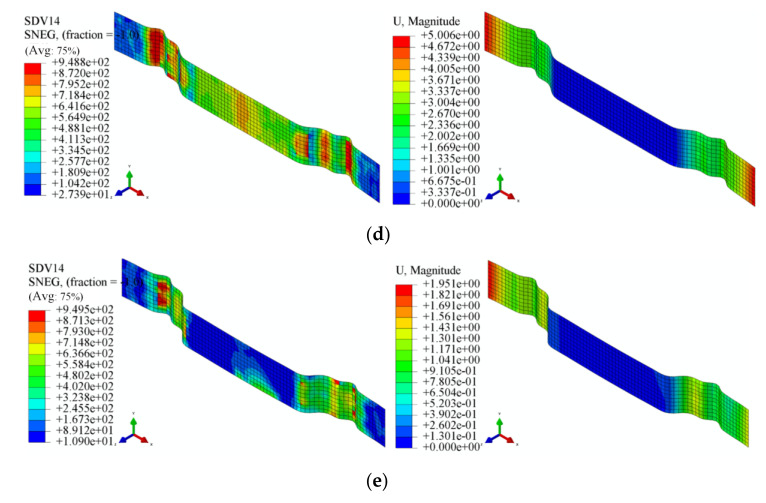
Local warm tensile bending springback test of U-shaped parts: (**a**) Test 1; (**b**) Test 2; (**c**) Test 3; (**d**) Test 4; and (**e**) Test 5.

According to the test parameters in Table 4, the finite element simulation was conducted to simulate the tensile bending springback. The results obtained from this simulation are shown in Figure 19. SDV14 signifies the Hill equivalent force computed for each grid of the sheet material, confirming the absence of fracture defects within the sheet. The variable U signifies the displacement exhibited by each grid of the sheet during the springback phase. The simulation results for the local warm forming test are shown in Figure 19a,d,e, with larger springback in the non-heating zone and smaller springback in the heating zone; the simulation results of the overall temperature forming test are shown in Figure 19b,c, with left–right symmetry of the displacements.

**Figure 20:**
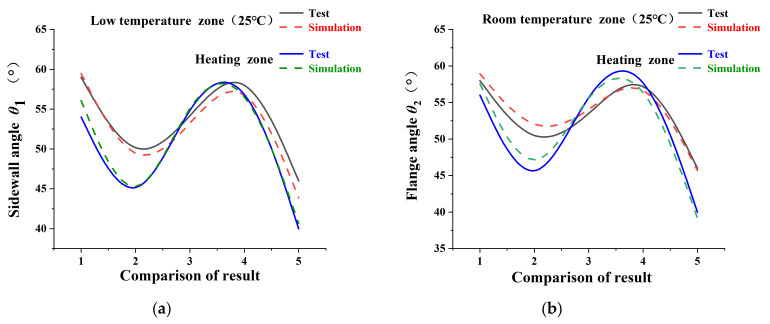
Comparison of local warm tensile bending springback test results and simulation results for U-shaped parts: (**a**) sidewall angle *θ*_1_; (**b**) flange angle *θ*_2_.

A comparative analysis of experimental and simulation results in Figure 20 reveals that the discrepancies between the simulated and measured springback for the U-shaped parts under local warm forming conditions are within 5%. By comprehensively evaluating Results 1, 4, and 5—specifically the simulated magnitudes of the sidewall angle (*θ*_1_) and flange angle (*θ*_2_) before and after localized heating—it is observed that both angles decrease post-heating, leading to reduced springback. This conclusively demonstrates the effectiveness of the proposed springback simulation method for advanced high-strength dual-phase steel (AHSS) in local warm forming applications.

### 4.3. Factors Analysis of Affecting Springback

**Figure 21:**
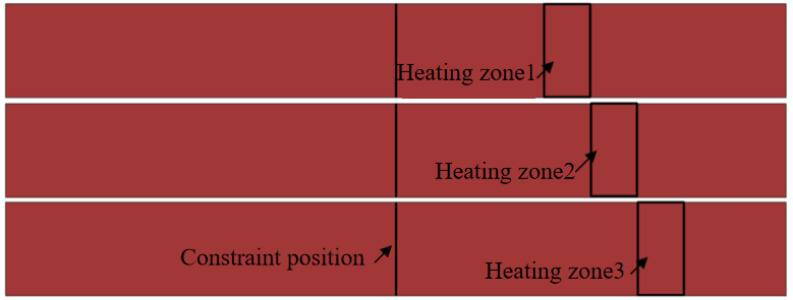
U-shaped parts bending springback heating zone.

Springback is influenced by multiple interacting factors with complex governing mechanisms. To analyze these influencing parameters in local warm forming springback, this study employed a cost-effective Box–Behnken experimental design, which requires fewer trials compared to full factorial approaches. For the U-shaped part local warm forming springback investigation, four critical factors were selected: blank holder force, heating temperature, heating zone, and punch radius. Regarding the heating zone configuration, the width was fixed at 15 mm. The horizontal positioning of the heating zone centerline—measured relative to the sidewall angle—was systematically varied at 0 mm, 15 mm, and 30 mm levels, as illustrated in Figure 21.

Considering the actual analyses in this paper, the cubic polynomial containing cross terms is chosen for the response surface prediction modeling, with the expression [31]:

(11) $$y=\beta_{0}+\sum_{i=1}^{k}\beta_{i}x_{i}+\sum_{i=1}^{k}\beta_{i}x_{i}^{2}+\sum_{i=1}^{k}\beta_{i}x_{i}^{3}+\sum_{i=1}^{k}\sum_{j=1}^{k}\beta_{ij}x_{i}x_{j}+\sum_{i=1}^{k}\sum_{j=1}^{k}\beta_{ij}x_{i}^{2}x_{j}$$

Combining the characteristics of the Box–Behnken test, the four factors mentioned above, with three levels selected for each factor, the design factor levels are shown in Table 5. The sidewall angle *θ*_1_ and flange angle *θ*_2_ are used as evaluation indexes in Table 5; *X*_1_ represents the blank holder force, *X*_2_ represents the heating temperature, *X*_3_ represents the heating zone, and *X*_4_ represents the punch radius. The response surface test was configured using DesignExpert software (Design Expert13) and executed through simulations in Abaqus, employing an established method for simulating local warm forming springback in advanced high-strength dual-phase steel. The results are shown in Table 6 after 29 finite element simulations.

The ANOVA results of the fitted models for the sidewall angle *θ*_1_ and flange angle *θ*_2_, obtained by screening terms not recommended for inclusion in the model using Design-Expert software (Design Expert13), are presented in Table 7 and Table 8, respectively. The response surface expressions relating the sidewall angle *θ*_1_ and flange angle *θ*_2_ to the process parameters are provided by Equations (12) and (13), respectively.

(12) $$\begin{array}{cc} \theta_{1} & =-63.25+0.5941X_{1}+0.2039X_{2}-0.04837X_{3}+8.107X_{4}-3.022\times 10^{-3}X_{1}X_{2}\\ & -0.004722X_{1}X_{3}-6.333\times 10^{-4}X_{1}X_{4}+2.353\times 10^{-3}X_{2}X_{3}-4.850\times 10^{-3}X_{2}X_{4}\\ & -0.02353X_{3}X_{4}+2.901\times 10^{-3}X_{1}^{2}-1.509\times 10^{-4}X_{2}^{2}-0.03039X_{3}^{2}-0.2581X_{4}^{2}\\ & -1.944X_{1}^{2}X_{2}-5.611\times 10^{-5}X_{1}^{2}X_{3}-2.667X_{1}^{2}X_{4}+2.335\times 10^{-6}X_{1}X_{2}^{2}\\ & +4.048\times 10^{-4}X_{1}X_{3}^{2}-1.658\times 10^{-6}X_{2}^{2}X_{3}+3.425X_{2}^{2}X_{4}-7.222\times 10^{-7}X_{2}X_{3}^{2} \end{array}$$

(13) $$\begin{array}{cc} \theta_{2} & =2.771+0.5941X_{1}-1.448X_{2}+0.5517X_{3}+4.377X_{4}-0.001867X_{1}X_{2}\\ & -0.01185X_{1}X_{3}+0.1343X_{1}X_{4}+4.16\times 10^{-4}X_{2}X_{3}+1.275\times 10^{-3}X_{2}X_{4}\\ & -7.4\times 10^{-3}X_{3}X_{4}+0.01593X_{1}^{2}-0.00009\times 10^{-5}X_{2}^{2}-0.01673X_{3}^{2}-0.413X_{4}^{2}\\ & -3.541X_{1}^{2}X_{2}+2.3\times 10^{-5}X_{1}^{2}X_{3}-1.133\times 10^{-3}X_{1}^{2}X_{4}+1.608\times 10^{-6}X_{1}X_{2}^{2}\\ & +2.60\times 10^{-4}X_{1}X_{3}^{2}-3\times 10^{-7}X_{2}^{2}X_{3}-9.25\times 10^{-7}X_{2}^{2}X_{4}+3.389\times 10^{-6}X_{2}X_{3}^{2} \end{array}$$

where *X*_1_ is the blank holder force, *X*_2_ is the heating temperature, *X*_3_ is the heating zone, and *X*_4_ is the punch radius.

After conducting an ANOVA (a crucial method for assessing the significance of the response surface model), it was determined that this analytical model accurately reflects the response relationship between the optimization objective and the design variables. By verifying the prediction accuracy of the fitted model, it was found that each test coefficient passed the test. Therefore, it can be concluded that the predicted values obtained from the fitted model are highly accurate and can be utilized for analysis in lieu of the actual model.

**Figure 22:**
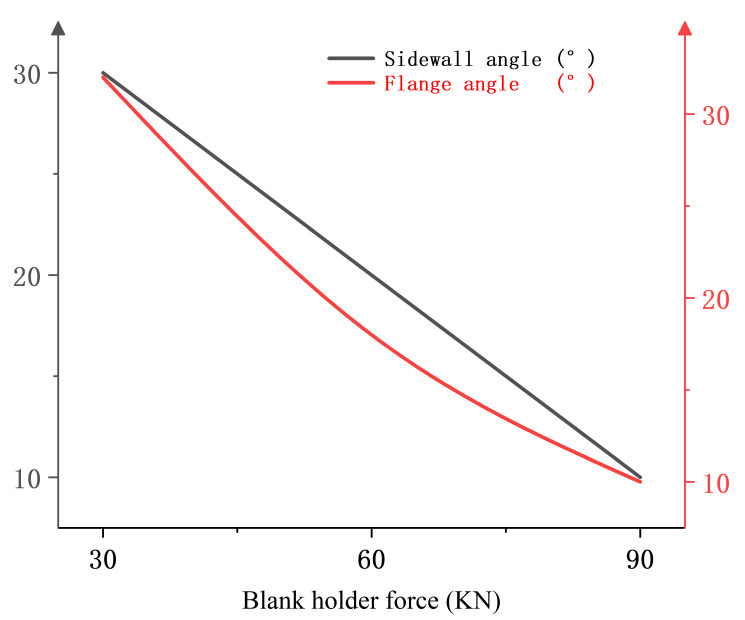
Single-factor relationship diagram between blank holder force and springback evaluation index.

Figure 22 shows the relationship between blank holder force and springback evaluation indexes when the heating temperature is 400 °C, the heating zone is 15 mm away from the punch-nose angle, and the punch radius is 7.5 mm. It can be found that the sidewall angle and flange angle decrease with the increase of the blank holder force, indicating that an increase in blank holder force is beneficial to inhibiting the springback behavior of the sheet.

**Figure 23:**
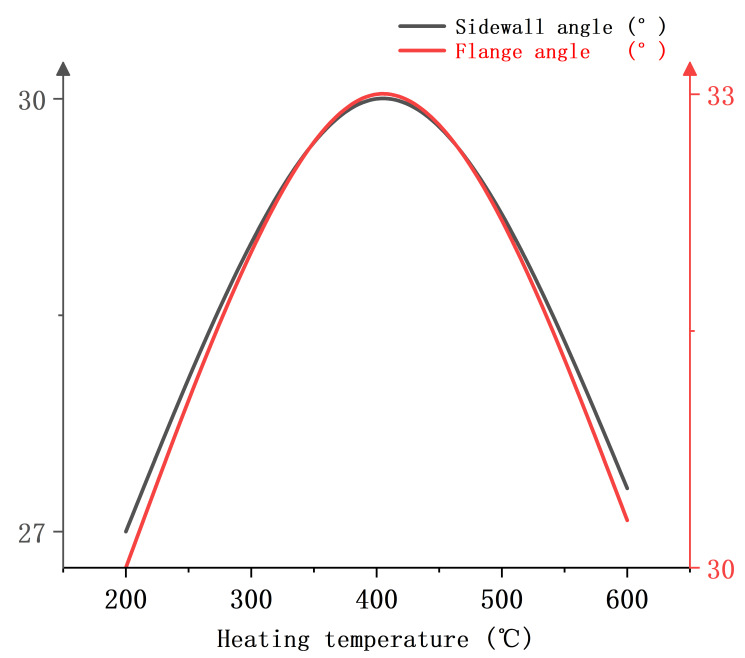
Single-factor relationship diagram between heating temperature and springback evaluation index.

Figure 23 shows the relationship between the heating temperature and springback evaluation indexes when the blank holder force is 30 kN, the heating zone is 15 mm away from the punch-nose angle, and the punch radius is 7.5 mm. It can be found that the heating temperature increases and the sidewall angle and flange angle show a tendency to increase and then decrease. The springback is largest when the heating temperature is 400 °C. The sidewall angle and flange angle at the heating temperature of 200 °C are smaller than those at the heating temperature of 600 °C.

**Figure 24:**
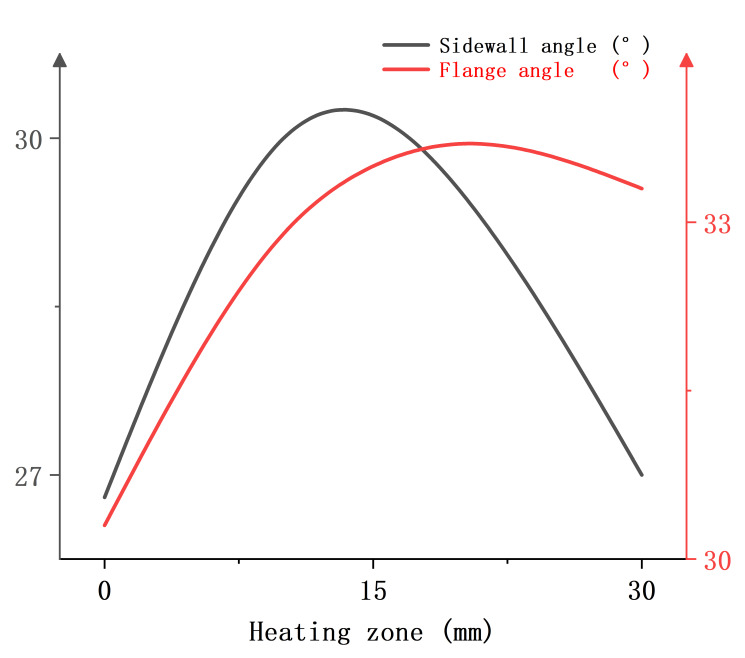
Single-factor relationship diagram between the position of the heating zone and the springback evaluation index.

Figure 24 shows the relationship between the heating zone and the springback evaluation indexes when the blank holder force is 30 kN, the heating temperature is 400 °C, and the punch radius is 7.5 mm. It can be found that as the heating zone moves away from the punch-nose angle, both the sidewall angle and the flange angle show a tendency to increase and then decrease.

**Figure 25:**
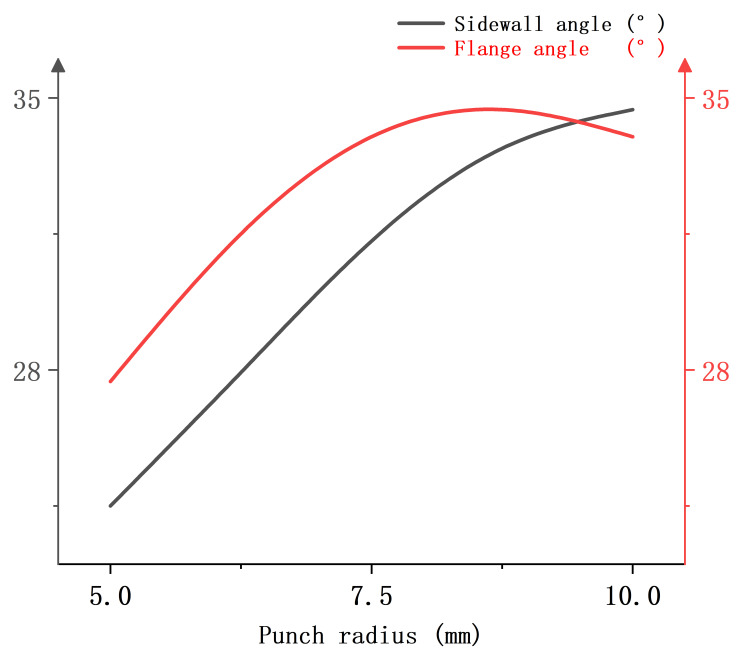
Single-factor relationship diagram between punch radius and springback evaluation index.

Figure 25 shows the relationship between the punch radius and the springback evaluation indexes when the edgewise force is 60 kN, the heating temperature is 400 °C, and the heating zone is 15 mm from the punch-nose angle. It can be found that the sidewall angle and flange angle increase with the increase of the punch radius, indicating that the decrease of the punch radius is conducive to the suppression of the springback behavior of the sheet material.

The study of the local warm forming of U-shaped parts shows that:

(1) The springback of the sheet can be suppressed by increasing the blank holder force and decreasing the punch radius, with the two factors exerting the most significant impact on the springback behavior.
(2) As the heating temperature increases, the springback volume of the plate initially enlarges and subsequently diminishes. This phenomenon is attributed to the occurrence of “blue brittleness” when the plate is heated. The “blue brittleness” enhances the strength of the sheet, leading to an increase in springback within the temperature range where this brittleness manifests.
(3) There is a higher springback when only one end of the feature zone is heated or when the temperature difference between the two ends of the feature is too large.

## 5. Conclusions

This study developed a thermo-mechanical coupled simulation algorithm for local warm forming, achieving a high-precision prediction of fracture and springback in DP780 U-shaped parts. The reliability of the algorithm was validated by the small discrepancy (<5%) between the experimental and simulation results. The response surface methodology quantitatively revealed the impact of process parameters on springback (e.g., an increased blank holder force significantly suppresses springback). Compared to existing studies, the core innovation lies in integrating heat transfer, mechanical deformation, and damage prediction into a unified framework, addressing the shortcomings of decoupled multi-physics models.

However, limitations remain. First, while the Hill’48 anisotropic criterion offers computational efficiency, its ability to describe anisotropic responses under complex stress paths is limited. Future work could adopt higher-order criteria to enhance universality. Second, the current algorithm’s applicability to ultra-high temperatures (>600 °C) or rapid cooling scenarios requires further validation, necessitating extended temperature ranges and phase transformation considerations. Additionally, although the algorithm is theoretically extendable to other dual-phase steels (e.g., DP980), the recalibration of constitutive parameters for different microstructures poses challenges for industrial standardization.

Future research should focus on:

(1) Adopting higher-order anisotropic constitutive models to enhance universality.
(2) Exploring synergistic processes combining local warm forming with additive manufacturing to advance lightweight, high-performance automotive components.

This research contributes to the industrial application of local warm forming technology for AHSS, offering a practical engineering value for achieving the cost-effective and high-quality production of complex geometries.

## Figures and Tables

**Table 1:** The chemical composition of DP780 and its mass fraction %.

C	Si	Mn	P	S	Alt
0.1	0.16	20.2	0.008	0.003	0.039

**Table 2:** *R*-values for the DP780 steel.

Steel	*r*_0_	*r*_45_	*r*_90_	$\bar{r}$
DP780	0.79	0.84	0.79	0.82

**Table 3:** Anisotropic parameters of dual-phase steel DP780.

*F*	*G*	*H*	*L*	*M*	*N*
0.56	0.56	0.44	1.5	1.5	1.5

**Table 4:** Process parameters for local temperature bending testing of U-shaped parts.

Serial Number	Punch Radius (mm)	Blank Holder Force (KN)	Stamping Depth (mm)	Heating Temperature (°C)
1	10	48	9	600
2	7	30	12	275
3	7	20	9	325 (Integral Heating)
4	10	40	9	378 (Integral Heating)
5	7	40	12	400

**Table 5:** Factor level table.

Level of Factors	Blank Holder Force *X*_1_ (KN)	Heating Temperature *X*_2_ (°C)	Heating Zone *X*_3_ (mm)	Punch Radius *X*_4_ (mm)
−1	30	200	0	5
0	60	400	15	7.5
1	90	600	30	10

**Table 6:** Box–Behnken test program and results of local warm forming springback of U-shaped parts.

Serial Number	*X*_1_ (kN)	*X*_2_ (°C)	*X*_3_ (mm)	*X*_4_ (mm)	*θ*_1_	*θ*_2_
1	30	200	15	7.5	27.09	30.75
2	90	200	15	7.5	16	9.76
3	30	600	15	7.5	28.1	31.47
4	90	600	15	7.5	14.31	7.43
5	60	400	0	5	13.34	5.81
6	60	400	30	5	12.84	7.8
7	60	400	0	10	25.29	18.83
8	60	400	30	10	21.26	19.71
9	30	400	15	5	23.77	27.58
10	90	400	15	5	6.3	1.45
11	30	400	15	10	34.41	35.19
12	90	400	15	10	15.79	8.57
13	60	200	0	7.5	21.58	15.22
14	60	600	0	7.5	21.28	15.31
15	60	200	30	7.5	16.73	15.61
16	60	600	30	7.5	17.62	17.07
17	30	400	0	7.5	28.73	30.12
18	90	400	0	7.5	15.53	8.38
19	30	400	30	7.5	24.33	33.31
20	90	400	30	7.5	12.37	9.32
21	60	200	15	5	13.48	5.85
22	60	600	15	5	14.08	6.29
23	60	200	15	10	24.59	18.1
24	60	600	15	10	24.71	18.6
25	60	400	15	7.5	21.23	15.48
26	60	400	15	7.5	21.23	15.48
27	60	400	15	7.5	21.23	15.48
28	60	400	15	7.5	21.23	15.48
29	60	400	15	7.5	21.23	15.48

**Table 7:** The analysis of variance results of the fitting model of the sidewall angle *θ*_1_.

Source	Sum of Squares	Degrees of Freedom	Mean Square	F-Value	*p*-Value	Result
Model	1038.71	22	47.21	8034.53	<0.0001	significant
A-A	325.62	1	325.62	55,411.93	<0.0001	highly significant
B-B	0.1296	1	0.1296	22.05	0.0033	
C-C	5.13	1	5.13	873.02	<0.0001	highly significant
D-D	103.73	1	103.73	17,652.72	<0.0001	highly significant
AB	1.82	1	1.82	310.14	<0.0001	highly significant
AC	0.3844	1	0.3844	65.41	0.0002	
AD	0.3306	1	0.3306	56.26	0.0003	
BC	0.354	1	0.354	60.25	0.0002	
BD	0.0576	1	0.0576	9.8	0.0203	
CD	3.12	1	3.12	530.13	<0.0001	highly significant
A^2^	1.59	1	1.59	271.38	<0.0001	highly significant
B^2^	1.03	1	1.03	175.88	<0.0001	highly significant
C^2^	14.26	1	14.26	2427.35	<0.0001	highly significant
D^2^	16.87	1	16.87	2871.59	<0.0001	highly significant
A^2^B	0.245	1	0.245	41.69	0.0007	
A^2^C	1.15	1	1.15	195.29	<0.0001	highly significant
A^2^D	0.0072	1	0.0072	1.23	0.3107	
AB^2^	15.71	1	15.71	2673.07	<0.0001	highly significant
AC^2^	14.93	1	14.93	2541.21	<0.0001	highly significant
B^2^C	1.98	1	1.98	336.95	<0.0001	highly significant
B^2^D	0.2346	1	0.2346	39.92	0.0007	
BC^2^	0.0021	1	0.0021	0.3595	0.5707	
Residual	0.0353	6	0.0059			
Lack of Fit	0.0353	2	0.0176			
Pure Error	0	4	0			
Cor Total	1038.74	28				

**Table 8:** The analysis of variance results of the fitting model of the flange angle *θ*_2_.

Source	Sum of Squares	Degrees of Freedom	Mean Square	F-Value	*p*-Value	Result
Model	2338.72	22	106.31	261.22	<0.0001	significant
A-A	695.64	1	695.64	1709.35	<0.0001	highly significant
B-B	0.2209	1	0.2209	0.5428	0.4891	
C-C	2.06	1	2.06	5.06	0.0655	
D-D	155.38	1	155.38	381.79	<0.0001	highly significant
AB	2.33	1	2.33	5.71	0.054	
AC	1.27	1	1.27	3.11	0.1283	
AD	0.06	1	0.06	0.1475	0.7142	
BC	0.4692	1	0.4692	1.15	0.3242	
BD	0.0009	1	0.0009	0.0022	0.964	
CD	0.308	1	0.308	0.7569	0.4177	
A^2^	153.37	1	153.37	376.86	<0.0001	highly significant
B^2^	0.894	1	0.894	2.2	0.1888	
C^2^	0.4301	1	0.4301	1.06	0.3436	
D^2^	43.22	1	43.22	106.2	<0.0001	highly significant
A^2^B	0.8128	1	0.8128	2	0.2073	
A^2^C	0.1985	1	0.1985	0.4876	0.5111	
A^2^D	13.01	1	13.01	31.96	0.0013	
AB^2^	7.45	1	7.45	18.31	0.0052	
AC^2^	6.16	1	6.16	15.14	0.0081	
B^2^C	0.0648	1	0.0648	0.1592	0.7037	
B^2^D	0.0171	1	0.0171	0.042	0.8443	
BC^2^	0.0465	1	0.0465	0.1143	0.7468	
Residual	2.44	6	0.407			
Lack of Fit	2.44	2	1.22			
Pure Error	0	4	0			
Cor Total	2341.16	28				

## Data Availability

The original contributions presented in this study are included in the article. Further inquiries can be directed to the corresponding author.

## References

[1] Yang X., Jia C., Zang L., Wang L., Li Z. (2019). Study on the formability of duplex steel with different strength grades. Sichuan Metall..

[2] Yan Y., Wang H., Li Q. (2015). The inverse parameter identification of Hill 48 yield criterion and its verification in press bending and roll forming process simulations. J. Manuf. Process..

[3] Ozturk F., Toros S., Kilic S. (2014). Effects of Anisotropic Yield Functions on Prediction of Forming Limit Diagrams of DP600 Advanced High Strength Steel. Procedia Eng..

[4] Hajbarati H., Zajkani A., Gholipour J. (2024). Temperature-dependent anisotropic hardening behavior of DP steels: A comparative study of Swift and Arrhenius-type models. Mater. Des..

[5] Xu Z., Shan D. (2018). Aluminum alloy sheet heat treatment and stamping integration technology in car body manufacturing. MST.

[6] Pepelnjak T., Kayhan E., Kaftanoglu B. (2019). Analysis of non-isothermal warm deep drawing of dual-phase DP600 steel. Int. J. Mater. Form..

[7] Li D. (2020). Simulation study on the formability of DP780 under high temperature conditions. Automob. Parts.

[8] Bielak R., Bammer F., Otto A., Stiglbrunner C., Colasse C., Murzin S.P. (2016). Simulation of forming processes with local heating of dual phase steels with use of laser beam shaping systems. Comput. Opt..

[9] Karaağaç İ., Kabakçi M.O., Başdoğan Z. (2023). The effects of laser-assisted heat treatment on warm formability of dual-phase materials. Ironmak. Steelmak..

[10] Zhou J., Yang X., Mu Y., Liu S., Wang B. (2021). Numerical simulation and experimental investigation of tailored hot stamping of boron steel by partial heating. J. Mater. Res. Technol..

[11] Wang K., Zhu B., Wang L., Wang Y., Zhang Y. (2018). Tailored properties of hot stamping steel by resistance heating with local temperature control. Procedia Manuf..

[12] Lee E.H., Yang D.Y., Yoon J.W., Yang W.H. (2015). Numerical modeling and analysis for forming process of dual-phase 980 steel exposed to infrared local heating. IJSS.

[13] Kim K.Y., Lee E.H., Park S.H., Kang Y.H., Park J.Y. (2020). An Infrared Local-Heat-Assisted Cold Stamping Process for Martensitic Steel and Application to an Auto Part. Metals.

[14] Küçüktürk G., Tahta M., Gürün H., Karaağaç I. (2022). Evaluation of the Effects of Local Heating on Springback Behaviour for AHSS Docol 1400 Sheet Metal. Trans. Famena.

[15] Sen N. (2020). Experimental investigation of the formability of ultrahigh-strength sheet material using local heat treatment. Ironmak.

[16] Boyu P., Fuhui S., Sebastian M. (2023). Constitutive modeling of temperature and strain rate effects on anisotropy and strength differential properties of metallic materials. Mech. Mater..

[17] Zhao H., Peng Y., Shi B. (2022). Research progress on anisotropic constitutive models of metallic materials. Plast. Eng..

[18] Zhang D. (2006). Rebound Theory and Finite Element Numerical Simulation of Sheet Metal Stamping Forming.

[19] Cui H., Li D., Fu Q., Lu Z., Xu J., Jiang N. (2023). Research on Forming Limit Stress Diagram of Advanced High Strength Dual-Phase Steel Sheets. Materials.

[20] Liu L., Li L., Liang Z., Huang M., Peng Z., Gao J., Luo Z. (2023). Towards ultra-high strength dual-phase steel with excellent damage tolerance: The effect of martensite volume fraction. Int. J. Plast..

[21] Liu L., Li L., He J., Liang Z., Peng Z., Gao J., Luo Z., Huang M. (2024). The unexpected low fracture toughness of dual-phase steels caused by ferrite/martensite interface decohesion. Scr. Mater..

[22] Xu Y., Dan W., Li C., Zhang W. (2020). Experimental Study of the Micromechanical Behavior of Ferrite in DP Steel Under Various Stress States. Met. Mater. Trans. A.

[23] Swift H.W. (1952). Plastic instability under plane stress. JMPS.

[24] Lu Z. (2024). Springback Simulation Method of Local Warm Forming for Advanced High-Strength Dual-Phase Steel Sheet.

[25] Sung J.H., Kim J.H., Wagoner R.H. (2010). A plastic constitutive equation incorporating strain, strain-rate, and temperature. IJP.

[26] Hajbarati H., Zajkani A. (2020). A novel finite element simulation of hot stamping process of DP780 steel based on the Chaboche thermomechanically hardening model. Int. J. Adv. Manuf. Technol..

[27] Luo M., Wierzbicki T. (2010). Numerical failure analysis of a stretch-bending test on dual-phase steel sheets using a phenomenological fracture model. IJSS.

[28] Bai Y., Wierzbicki T. (2007). A new model of metal plasticity and fracture with pressure and Lode dependence. IJP.

[29] Bai Y., Wierzbicki T. (2010). Application of extended Mohr-Coulomb criterion to ductile fracture. Int. J. Fract..

[30] Li Y., Li D., Song H., Wang Y., Wu D. (2024). Temperature Dependency of Modified Mohr-Coulomb Criterion Parameters for Advanced High Strength Dual-Phase Steel DP780. Metals.

[31] Tao Y. (2016). Research on the Optimization of Back-Arc Hot Stamping Process and Springback Compensation of Large Hollow Blades.

